# Functional Network Changes After High-Frequency rTMS Over the Most Activated Speech-Related Area Combined With Speech Therapy in Chronic Stroke With Non-fluent Aphasia

**DOI:** 10.3389/fneur.2022.690048

**Published:** 2022-02-10

**Authors:** Won Kee Chang, Jihong Park, Ji-Young Lee, Sungmin Cho, Jongseung Lee, Won-Seok Kim, Nam-Jong Paik

**Affiliations:** Department of Rehabilitation Medicine, Seoul National University College of Medicine, Seoul National University Bundang Hospital, Seongnam-si, South Korea

**Keywords:** transcranial magnetic stimulation, stroke, aphasia, neuronal plasticity, functional neuroimaging

## Abstract

**Objective:**

High-frequency repetitive transcranial magnetic stimulation (HF-rTMS) to the lesional hemisphere requires prudence in selecting the appropriate stimulation spot. Functional near-IR spectroscopy (fNIRS) can be used in both selecting the stimulation spot and assessing the changes of the brain network. This study aimed to evaluate the effect of HF-rTMS on the most activated spot identified with fNIRS and assess the changes of brain functional network in the patients with poststroke aphasia.

**Methods:**

A total of five patients received HF-rTMS to the most activated area on the lesional hemisphere, followed by 30 min of speech therapy for 10 days. The Korean version of the Western aphasia battery (K-WAB) and fNIRS evaluation were done 1 day before the treatment, 1 day and 1 month after the last treatment session. Changes of K-WAB and paired cortical interaction and brain network analysis using graph theory were assessed.

**Results:**

Aphasia quotient in K-WAB significantly increased after the treatment (*P* = 0.043). The correlation analysis of cortical interactions showed increased connectivity between language production and processing areas. Clustering coefficients of the left hemisphere were increased over a sparsity range between 0.45 and 0.58 (0.015 < *p* < 0.031), whereas the clustering coefficients of the right hemisphere, decreased over a sparsity range 0.15–0.87 (0.063 < *p* < 0.095). The global efficiency became lower over a network sparsity range between 0.47 and 0.75 (0.015 < *p* < 0.063).

**Conclusion:**

Improvement of language function and changes of corticocortical interaction between language-related cortical areas were observed after HF-rTMS on the most activated area identified by fNIRS with combined speech therapy in the patients with poststroke aphasia.

## Introduction

Aphasia is one of the most common consequences of stroke. The incidence of aphasia has been reported variously, and the proportion of patients experiencing severe aphasia has been reported up to 12% even at 6 months after stroke (1–3). In the poststroke aphasia rehabilitation, repetitive transcranial magnetic stimulation (rTMS) is used to reinforce the activity of the brain regions in the language-associated cortex and has provided the opportunity to improve the language function (4). Most studies have chosen the low-frequency rTMS (LF-rTMS) on the contralesional homologous area to improve language function after stroke (5–8). However, observational studies have reported a doubtful role of the right hemisphere and interhemispheric inhibition in the recovery of non-fluent aphasia (9, 10) and have highlighted the role of the left hemisphere since the patients with better recovery from aphasia showed higher activation in the left hemisphere (11). Based on these findings, there have been attempts to apply high-frequency rTMS (HF-rTMS) to the lesional and perilesional areas to improve language functions (12–14). Application of HF-rTMS to the affected cerebral hemisphere requires prudence in the selection of appropriate stimulation site (15) and identification of the activated areas during the language processing often needs the usage of functional imaging techniques (16). Due to the additional effort required in those procedures, HF-rTMS on improving aphasia is not actively adopted in the clinical field yet and the reports on the effect of the excitatory stimulation on the lesional hemisphere are scarce (17).

Functional near-IR spectroscopy (fNIRS) has become a promising imaging technique for functional brain research recently (18, 19). fNIRS has several advantages such as instrumental portability, reliable signal acquisition during motion, and high temporal sampling rate compared to functional MRI (fMRI). Although the spatial resolution of fNIRS is relatively lower than fMRI (20), several previous studies have proven that fNIRS also can offer a valid capacity of measurement or localization when it is applied to the specific cortical region. Thus, it is widely used in the localization of brain activation and the identification of functional connectivity during different states in both normal and patients with different pathological conditions (21–24) in the clinical settings. Furthermore, since fNIRS can detect changes in the cortex which is the target of neuromodulation, the stimulation site of rTMS can be selected using the results of fNIRS.

The purpose of this study was to investigate whether improvement of language function can be achieved by applying lesional HF-rTMS on the most activated area detected using fNIRS combined with the speech therapy and to identify the differences in the brain functional networks and the changes in the functional connectivity after HF-rTMS in the patients with chronic stable poststroke non-fluent aphasia.

## Methods

### Participants

A total of five patients with chronic stroke with non-fluent aphasia over 6 months after stroke satisfied the following criteria were included in this study: (a) right-hand dominant confirmed with Edinburgh handedness inventory; (b) first-ever stroke affecting left hemisphere indicated by MRI lesion; (c) presence of non-fluent aphasia on the Korean version of the Western aphasia battery (K-WAB) (25); (d) native speaker of Korean language; and (e) provision of written informed consent. Patients with age <18 years, underlying degenerative or metabolic disorder or supervening medical illness, any contraindication to MRI (i.e., intracranial metal implants and claustrophobia), or any contraindication to rTMS (e.g., seizures or epilepsy) were excluded.

### Study Procedures

**Figure 1:**
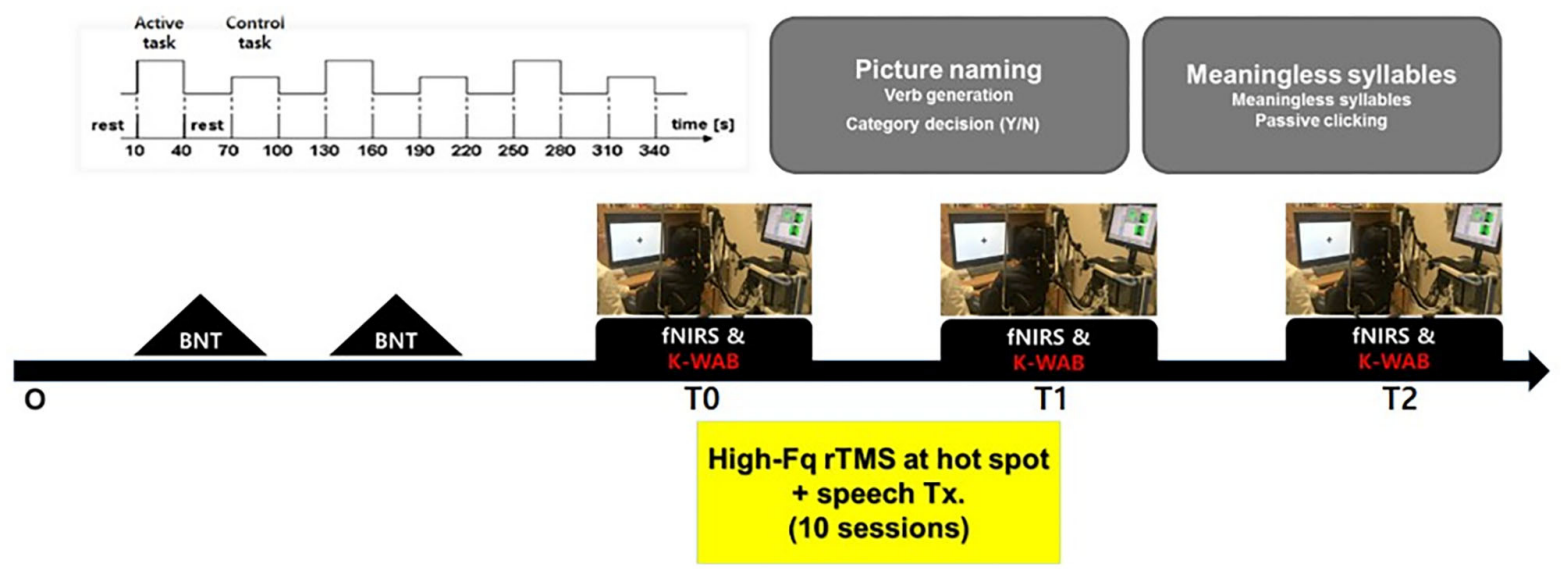
Schematic drawing of the study procedures. BNT, Boston naming test; fNIRS, functional near-IR spectroscopy; K-WAB, Korean version of the Western aphasia battery.

The Korean version of the Boston (K-BNT) naming test (26) was performed twice at an interval of 2 weeks, to confirm that the participants were in the chronic state of non-fluent aphasia. K-WAB and fNIRS evaluation were done 1 day before (T0), 1 day after 10 sessions of treatment (T1), and 1 month after the last treatment session (T2). Participants performed sequential tasks using a block design paradigm consisting of repeated picture naming and rest during fNIRS measurement. The stimulation (100% of resting motor threshold, 10 Hz, 800 stimuli, and intertrain interval every 200 pulses) was delivered with rTMS system (Supplementary Figure 1) consisting of the TMS equipment (MagPro, MagVenture A/S, Farum, Denmark) and a figure-8 shape coil with a 75 mm outside diameter (MCF-B65, MagVenture A/S, Farum, Denmark) to the most activated channel in the Broca, Wernicke, and adjacent area on T0 fNIRS measurement. The most activated channel on the fNIRS was matched to the MRI-guided navigation TMS system (Brainsight, Rogue Research, Montreal, Canada) by matching the channel to the curvilinear coordinates using an optical digitizer (Polaris Vicra, NDI, Ontario, Canada). The TMS coil was applied tangentially to the scalp of the area. The participants received HF-rTMS using the navigation rTMS system in combination with speech therapy (30 min per day) including prompting verbal response and naming, cueing hierarchy training of naming, word pair repetition, and reading comprehension for 10 consecutive weekdays. The speech therapy was done by a single Speech-Language Pathologist. We used the same method as described previously for the determination of the resting motor threshold and the application of the navigation rTMS system (27). Written informed consent was obtained from the subjects and the schematic flow of the study is given in Figure 1. This study was approved by the Institutional Review Board of Seoul National University Bundang Hospital (B-1507/308-006).

### fNIRS Acquisition

**Figure 2:**
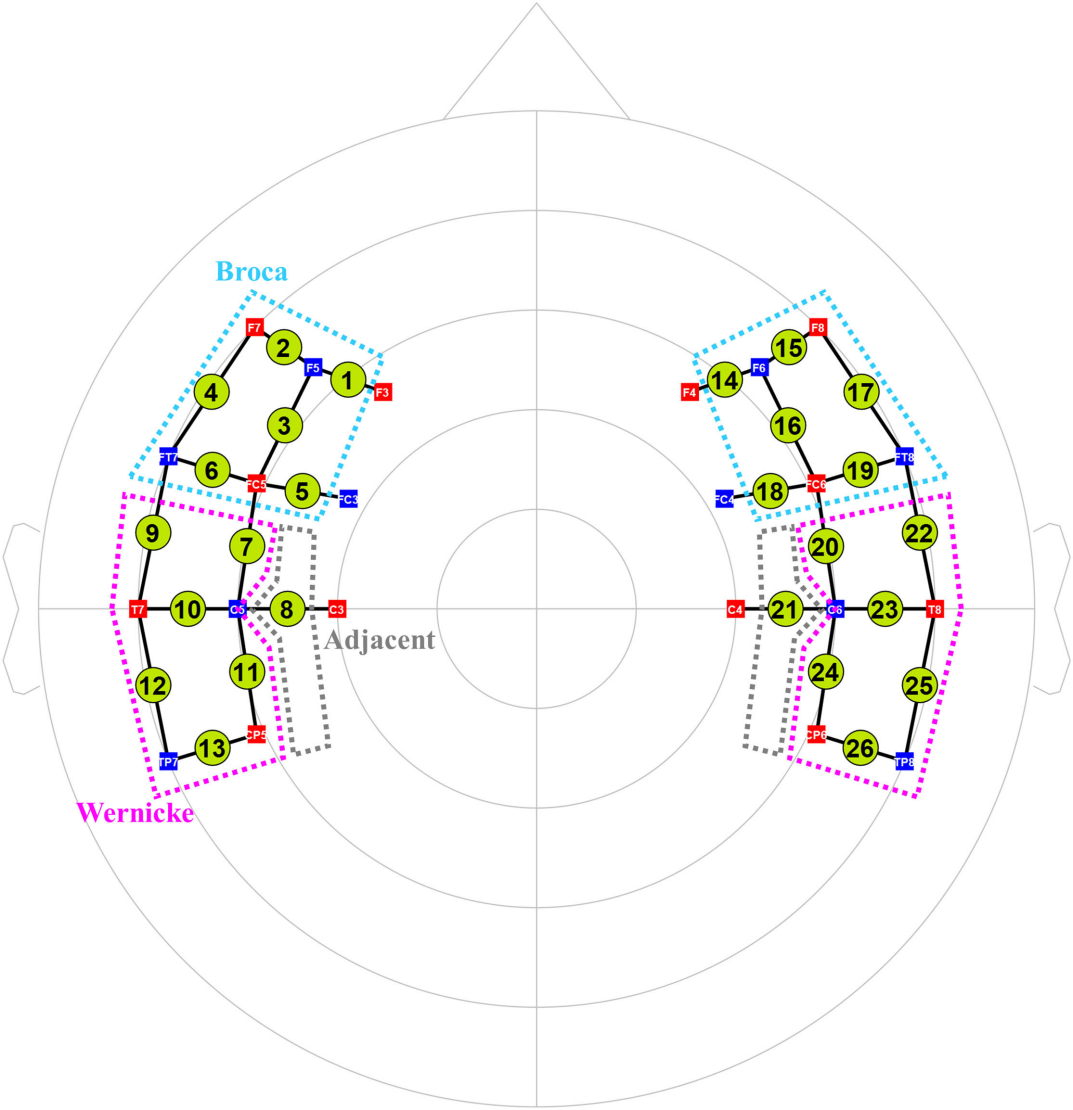
The arrangement of 26 fNIRS channels (12 sources and 10 detectors). Total 26 channels were formed by positioning 12 sources and 10 detectors with a source-detector separation of 3 cm covering Broca (cyan dotted lines) and Wernicke (pink ones) and adjacent (gray ones) areas based on the International 10–20 electroencephalography system. Green circles indicate the 26 channels with the numbers being the channel IDs. Red squares indicate the sources; blue squares indicate the detectors. The white characters in the squares mean the positions of the sources and detectors on the 10–20 system.

We used an fNIRS system (NIRx^®^ Medizintechnik GmbH, Berlin, Germany) with light-emitting diodes of 760 and 850 nm wavelengths to assess cortical activity. We set 26 channels by arranging 12 sources and 10 detectors at intervals of 3 cm and positioned them across Broca, Wernicke, and adjacent perilesional areas according to the International 10–20 electroencephalography (EEG) system (Figure 2). Three different sizes of textile EEG caps (Easycap^®^, Herrsching, Germany) with circumferences of 54, 56, and 58 cm were used to mount the optodes on the heads of subjects according to head circumference. Changes in oxygenated hemoglobin (oxyHb), deoxygenated hemoglobin (deoxyHb), and total hemoglobin concentration in the cortex were detected by applying the modified Beer–Lambert law (28). There was an initial rest block for 2 min, followed by six task blocks alternated with six control blocks that lasted for 30 s. Subjects were instructed to sit comfortably on a chair and to remain motionless with eyes open for the rest blocks. During the following task blocks, subjects were instructed to name the pictures (overt generation) from the picture database of the K-BNT which were presented for 2 s at the center of a 15-inch LCD screen. During the control blocks, subjects were instructed to name the days of the week (from Monday to Sunday) slowly.

### fNIRS Data Preprocessing

Optical density data were processed with NIRSLAB software version 14.0 (NIRx Medizintechnik GmbH, Berlin, Germany) (29). The differential path lengths were set as 7.25 and 6.38 for wavelengths of 760 and 850 nm, respectively (30), for detecting the changes in the concentration of oxyHb, deoxyHb, and total hemoglobin. OxyHb, which is most sensitive to changes in cortical blood flow, was used in the analysis because deoxyHb shows large interpersonal variation in task-related changes (31). Artifacts due to discontinuity and spikes were removed manually. All data sets were filtered with a 0.01-Hz high-pass filter and a 0.2-Hz low-pass filter to remove instrumental or physiological noise (32).

### Analysis of fNIRS Data

To find out the most significantly activated channel during the language performance as a HF-rTMS target site, general linear model (GLM)-based statistical data analysis was performed using Statistical Parametric Mapping 8 (SPM, version 8) embedded in the nirsLAB program. For each patient, GLM coefficients of an oxyHb signal of a channel were calculated with the canonical hemodynamic response function (HRF) after the oxyHb signal was filtered with a temporal filter of discrete cosine transform function and pre-colored with HRF. Then, a SPM t-image was obtained between the task and control blocks and the absolute values of all *t*-statistic values were calculated (*p* < 0.05). Finally, the channel with the largest absolute value of *t*-statistic value was selected for the HF-rTMS target site. The *p*-value was incremented by 0.001 until a channel is detected if no channel was found with *p* < 0.05.

Task-related connectivity and brain network analysis by using graph theory were analyzed with the language processing and production cortices and adjacent regions. The channels for those cortical regions were identified and localized, based on the International 10–20 EEG System.

The Pearson's correlation coefficient was used to determine the strength of functional connectivity in pairwise relationships between any pair of channels from the processed signals from 26 channels. Before any statistical analysis, correlation values were converted to Fisher *z*-values through Fisher *z* transformation (33) to reduce statistical bias caused by sampling distribution (34). Whole-brain connectivity analysis aimed to construct a functional connectivity map between channels and to identify the longitudinal changes using *z*-scores. Connections with correlation coefficients greater than the predefined threshold, *z* = 1.0, were arbitrarily defined as strong connectivity.

The longitudinal changes in neural networks in each hemisphere were analyzed with graph theory, and all 26 channels were included in this analysis. We focused on two graph-theoretical metrics using the brain connectivity toolbox: (35) global efficiency and clustering coefficient. The global efficiency is inversely related to the distance between nodes and is typically known to be an indicator of parallel information transfer and integrated processing (36). The clustering coefficient is a measure of how closely neighboring nodes are connected to each other, considering the environment adjacent to each node (37). All metrics were obtained across a whole range of sparsity.

### Statistical Analysis

All the statistical analyses were performed with SPSS version 21.0 (IBM Corporation, Armonk, New York, USA) and MATLAB and Statistics Toolbox version 2012b (MathWorks Incorporation, Natick, Massachusetts, USA). In all the cases, statistical significance was defined as *p* < 0.05, except for the results from the network analysis using graph theory where *p* < 0.1 was used.

## Results

**Figure 3:**
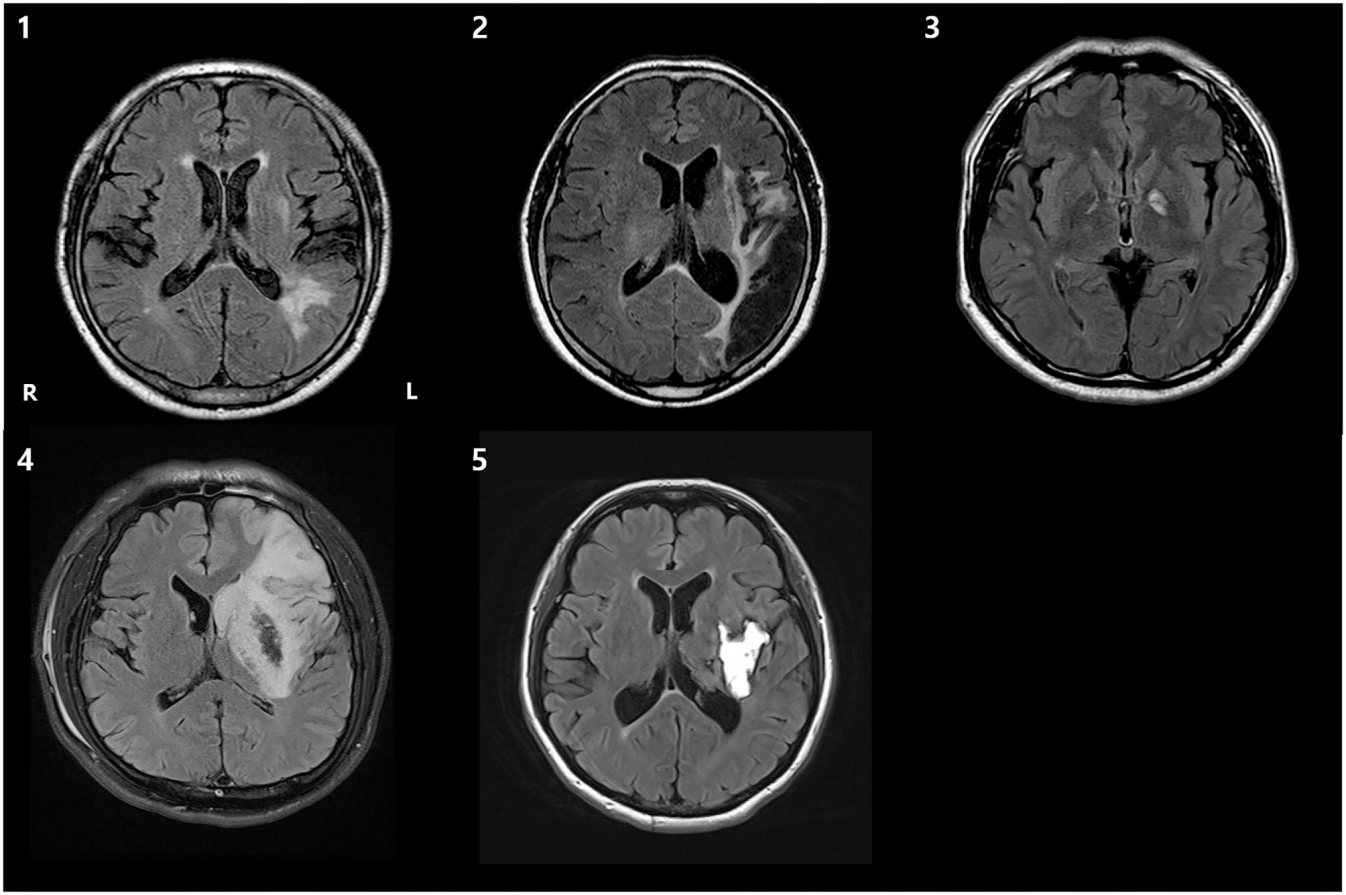
T2 fluid-attenuated inversion recovery (FLAIR) axial brain MRI images of 5 patients, demonstrating the lesion. R, right; L, left.

The clinical, demographic data and MRI scans of five patients are shown in Table 1 and Figure 3. The scores of 1st and 2nd K-BNT performed before initial fNIRS and K-WAB evaluation (T0) suggested that all the patients were in a chronic stage of poststroke aphasia.

**Table 1:** Clinical and demographic data.

**Patient number**	**1**	**2**	**3**	**4**	**5**
Age (years)	49	67	45	60	60
Gender	Male	Female	Male	Male	Female
Stroke type	Infarction	Infarction	Infarction	Infarction	Hemorrhage
Lesion side	Left	Left	Left	Left	Left
Lesion location	Middle cerebral artery territory	Middle cerebral artery territory	Basal ganglia	Middle cerebral artery territory	Basal ganglia
Time since stroke (months)	10	72	23	24	73
Dominant hand	Right	Right	Right	Right	Right
K-BNT (pre 1st)	7/3/14	0/1/6	57/2/1	0/0/8	25/3/17
K-BNT (pre 2nd)	7/3/14	0/0/6	59/0/1	0/0/8	25/3/17
K-WAB (T0)					
Speech	13	7	19.5	8	14.5
Repeat	16	16	100	60	22
Reading	59	45	100	29	54
Comprehension	142	89	197	96	146
Naming	43	7	94	51	78
Writing	45	18	99	28	36
AQ	52.0	27.6	97.6	47.8	63.6
LQ	53.9	25.0	98.4	40.1	57.1

*K-BNT, Korean version of the Boston naming test; K-WAB, Korean version of the Western aphasia battery; AQ, aphasia quotient; LQ, language quotient*.

*K-BNT score indicates naming without help score/naming with sematic help score/naming with phonemic help score, respectively*.

**Figure 4:**
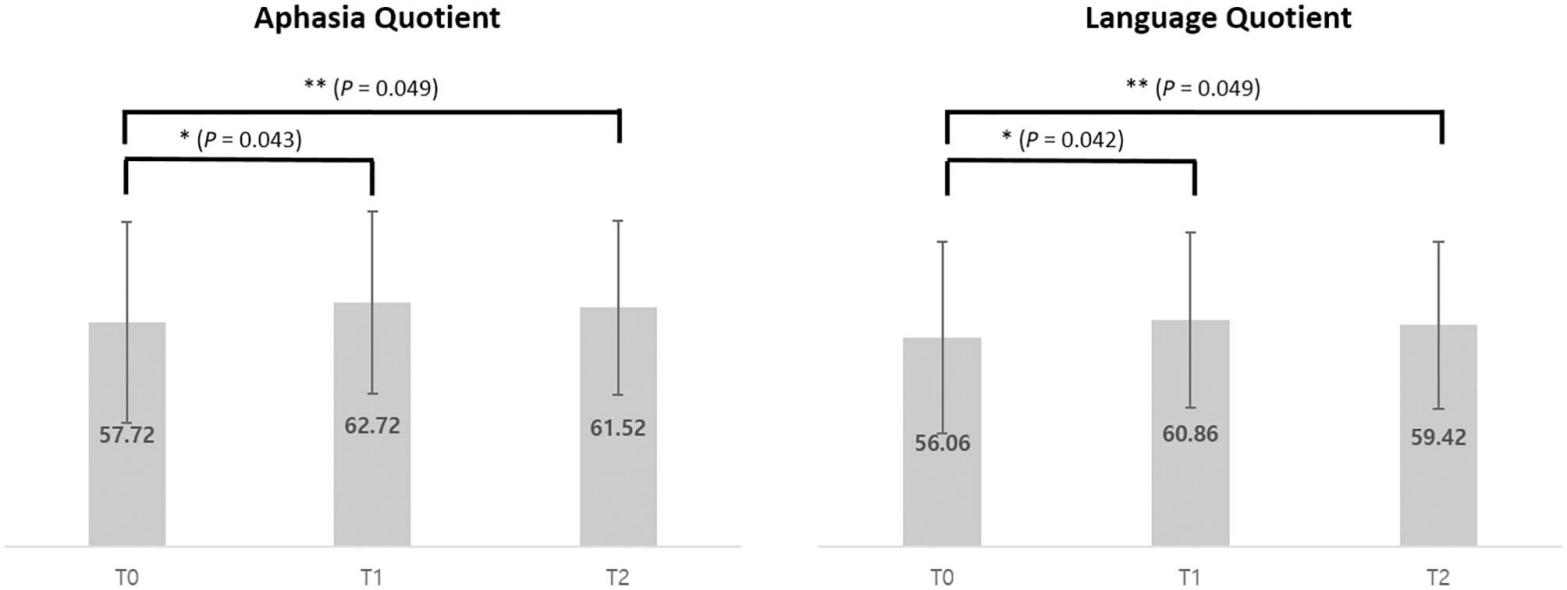
Average of Aphasia Quotient and Language Quotient scores of pre (T0), post (T1), and 1 month after (T2) intervention of 5 participants. *p < 0.05 (using Wilcoxon signed-rank test). **p < 0.05 (using Friedman test).

The K-WAB scores are shown in Table 2 and the aphasia quotient (AQ) and language quotient (LQ) in the K-WAB increased after HF-rTMS combined with speech therapy in all the participants at T1 (see Supplementary Table 1 for subsection scores). There was significant difference in both AQ (*p* = 0.049) and LQ (*p* = 0.049) across timeline. Pair-wise analysis showed significant increase of both AQ (*p* = 0.043) and LQ (*p* = 0.042) between T0 and T1 but not between T1 and T2 (Figure 4).

**Table 2:** Result from K-WAB in five participants.

**Patient number (Stimulation Site)**	**1 (Broca)**	**2 (Adjacent)**	**3 (Wernicke)**	**4 (Wernicke)**	**5 (Wernicke)**
fNIRS channel No.	5	8	10	10	11
**Aphasia Quotient**					
T0	52.0	27.6	97.6	47.8	63.6
T1	56.8	34.4	99.0	56.4	67.0
T2	55.6	38.4	99.0	55.6	59.0
**Language Quotient**					
T0	53.9	25.0	98.4	40.1	57.1
T1	58.4	33.1	99.5	51.7	61.6
T2	57.8	35.4	99.5	50.1	54.3

*The results of K-WAB at each time point in 5 subjects with chronic non-fluent aphasia*.

*The scores in K-WAB have increased after HF-rTMS combined with speech therapy in all the subjects. The activated channel of the left hemisphere in each patient was selected as the stimulation site for HF- rTMS*.

**Figure 5:**
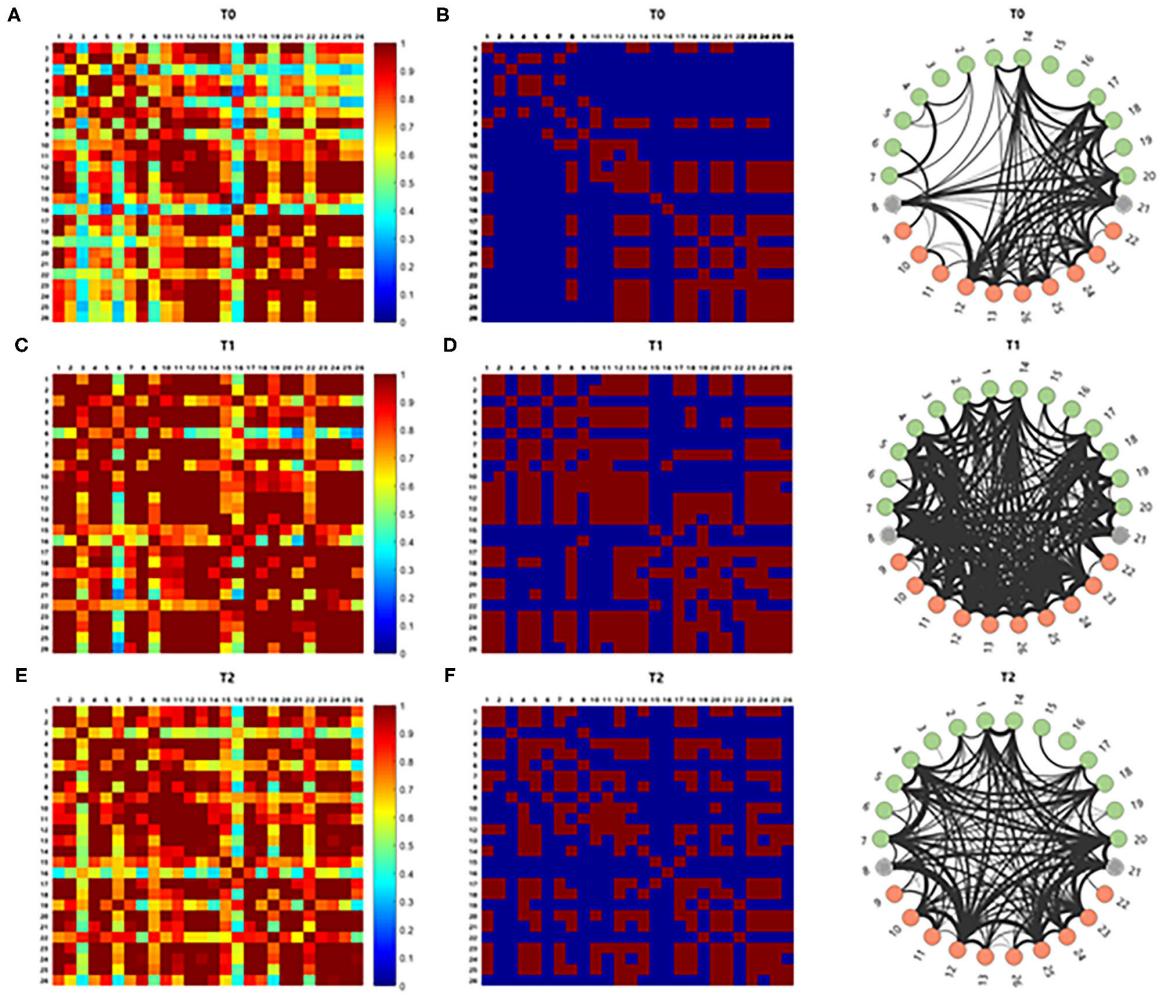
The brain connectivity between paired channels by using correlation analysis was performed and indicated by a different color according to the intensity of the correlation **(A,C,E)**. Stronger cortical interactions with threshold *z* > 1.0 were represented by line according to thickness **(B,D,F)**. Those with *z* > 1.0 comprise 15.866% of the total interactions. The correlation analysis for comparison of the cortical interaction in the cortex showed increased strength of connectivity in the left hemisphere at T1 and T2 compared to T0.

The correlation analysis for comparison of the average cortical interactions in the cortex in five participants showed increased strength of connectivity in the language-related cortical areas at T1 and T2 compared to T0. Cortical interactions with a strong threshold (*z* > 1.0) are represented by lines, with thicker lines representing stronger cortical interactions (Figure 5).

For the network analysis, the correlation matrices of the five subjects were thresholded into the hemispheric brain network at each time point over the whole range of sparsity from 0 to 1. Parameters such as global efficiency and clustering coefficient from graph theoretical network analysis of each hemisphere at each time point are profiled as sparsity functions.

**Figure 6:**
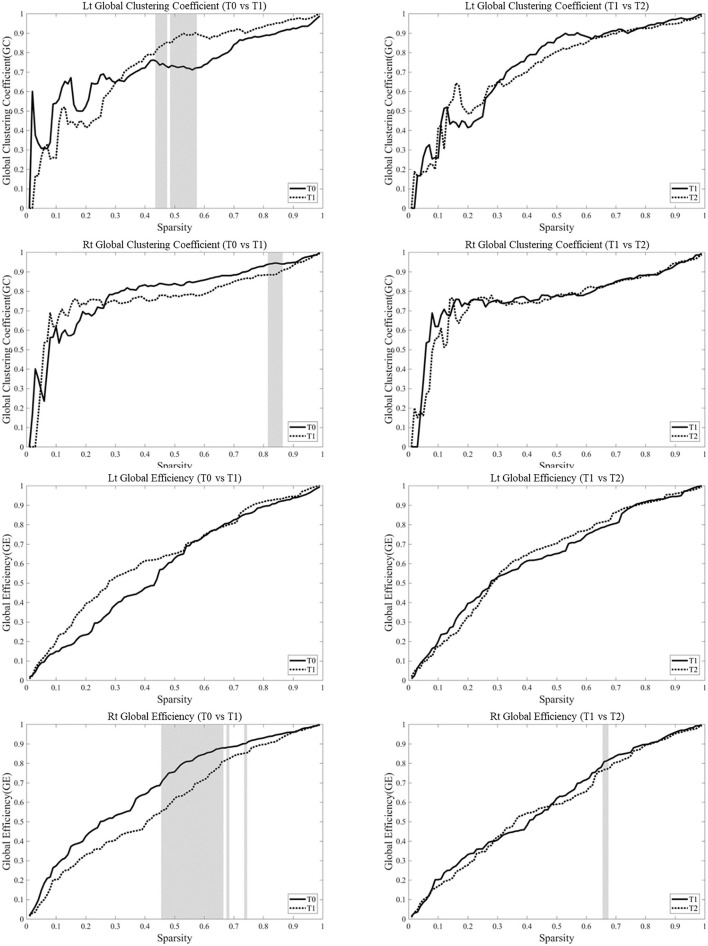
Network parameters were based on the task-based functional networks generated from the measurements of changes in oxyhemoglobin (OxyHb) over the whole range of sparsity. Analysis of the changes in parameters from T0 to T1 in each hemisphere showed that the clustering coefficients of the left hemisphere at T1 was higher than those at T0 over certain sparsity ranges between 0.45 and 0.58 (0.015 < *p* < 0.031), whereas the clustering coefficient in the right hemisphere became lower at T1 than those at T0 over certain sparsity ranges between 0.15 and 0.87 (0.063 < *p* < 0.095). The clustering coefficient at T1 and T2 was not different in bilateral hemispheres. The global efficiency became lower at T1 over certain sparsity ranges between 0.47 and 0.75 (0.015 < *p* < 0.063). The gray zone represents significant difference (*p* < 0.1).

Analysis of the changes in parameters from T0 to T1 in each hemisphere showed that the clustering coefficients of the left hemisphere at T1 was higher than those at T0 over certain sparsity ranges between 0.45 and 0.58 (0.015 < *p* < 0.031), whereas the clustering coefficient in the right hemisphere was lower at T1 than those at T0 over certain sparsity ranges between 0.15 and 0.87 (0.063 < *p* < 0.095). The clustering coefficient at T1 and T2 was not different in bilateral hemispheres. The global efficiency became lower at T1 over certain sparsity ranges between 0.47 and 0.75 (0.015 < *p* < 0.063) (Figure 6).

## Discussion

This study reports improvement of aphasia by delivering HF-rTMS at the most activated lesional area determined with fNIRS followed by speech therapy in patients with chronic poststroke aphasia. In some patients, the effect lasted till 1 month after the therapy. The connectivity between the language areas was fortified as well as the clustering coefficient of the left hemisphere increased after the HF-rTMS.

Patients with poststroke aphasia may have maladaptive cortical changes in both cerebral hemispheres including the imbalance of the hemispheres (38, 39) and rTMS is expected to normalize the patterns of neuronal activation by correcting this imbalance. Most previous studies have applied the low-frequency stimulation at 1 Hz, on the inferior frontal gyrus of the contralesional hemisphere (40–44) and a meta-analysis reported that this inhibitory protocol had a positive effect on the recovery of poststroke aphasia, although its long-term effect was not confirmed (45). On the other hand, studies reporting positive results with excitatory (or high frequency) stimulation on the lesional language-associated area are scarce (12–14). The difficulty of determining stimulation spot on the lesional hemisphere might be one reason. Reorganization of language circuit in the lesional hemisphere often occurs after stroke and to ensure the stimulation is administered to the intact area of the language circuit instead of the damaged enphalomalacic area, a functional measurement tool is required (15). In this study, we have chosen fNIRS as our tool for detecting the most activated “hot spot” because of its high availability in the clinical settings (46). To the best of our knowledge, this is one of the first studies to explore the feasibility of using fNIRS with HF-rTMS on the lesional side in patients with poststroke aphasia.

Of the five patients, one patient showed the most activated spot in the Broca area, another patient showed the most activated spot in the perilesional area, and the other three patients showed the Wernicke area. There was no significant difference in the demographic variables or degree of improvement in K-WAB score from T0 to T1 between the subgroups. However, since there were only five patients, further experiments with a much larger sample are needed to determine whether there is difference in the degree of improvement between stimulation sites.

Patient no. 2 exhibited the most activated spot in Channel 8, the primary somatosensory cortex, which is not a typical language-associated cortex. There have been articles reporting perilesional adjacent cortical activations in patients with poststroke aphasia (47, 48). She had a large chronic infarction involving the left fronto–temporo–parietal lobe with relative sparing of the primary sensory cortex (Supplementary Figure 2). Thus, we considered Channel 8 as a valid target for HF-rTMS stimulation. One patient (patient no. 5) exhibited a significant decline of both AQ and LQ scores from T1 to T2 although she did not complain of a subjective decrease in her language functions between the period. It is unclear why she did not benefit from HF-rTMS in the long term, however, similar findings have been reported in the studies with combined non-invasive brain stimulation and speech therapy (14, 49).

Whole-brain correlation analysis with the average *z*-scores between all the paired channels in the language production and processing areas in the five participants suggested that there might be a globally impaired corticocortical interaction, especially between lesional and contralesional hemispheres. A *z*-score of 1.00, which was equal to the upper 15.87%, was arbitrarily selected as the threshold for strong cortical interactions to compare the approximate tendency of the correlation. After lesional HF-rTMS combined with speech therapy, there were more strong paired cortical interactions between language areas and hemispheres.

We also performed brain network analysis using graph theory to explore the changes and differences in the language areas in each hemisphere. The graph theory provides a tool for deriving the global properties of complex brain networks, such as the efficiency of structures and clustering of nodes (36). Our results are distinctive in the sense that we showed different changes in the lesional and contralesional hemispheres after lesional HF-rTMS combined with speech therapy in the network parameters including global efficiency and global clustering coefficient. The most notable finding was the decreased global efficiency and global clustering coefficient in the contralesional hemisphere while the global clustering coefficient in the lesional hemisphere increased during a certain sparsity range. Brain reaction after an acute injury or slowly progressive neurodegenerative disease is known to be associated with a shift of the brain network toward a random network, which is possibly related to axonal sprouting (50, 51). The random axonal outgrowth after stroke could be related to a less optimal network and the optimal strategy should contain both reorganization of the large-scale network to transfer the information faster and increasing the power of local processing (52). However, how the language-related brain network associated in dominant and non-dominant hemispheres recovers needs further scrutinization. The global clustering coefficient measures how many neighbors of a node are connected to each other, and the global efficiency that is inversely related to path length is the average number of edges needed to move from one node to another in the network (53). The decreased global clustering coefficient and global efficiency in the contralesional hemisphere and the increased global clustering coefficient in a certain sparsity range with the improved language function after HF-rTMS combined with speech therapy could be one of the features of proper reorganization in the language network. Our findings are congruent with previous studies that investigated changes in functional activities on the lesional hemisphere. Szaflarski et al. have demonstrated increased blood flow in the left Broca and Wernicke area after applying HF-rTMS to the left Broca area (12) and Ulm et al. have reported increased activities in the left Broca and occipital area in the patients who received anodal-transcranial direct current stimulation to the left hemisphere (54). On the other hand, Hara et al. reported application of HF-rTMS to the lesional hemisphere resulted in increased activity in the contralesional hemisphere (55), therefore further studies are warranted to conclude the changes of functional activities after application of HF-rTMS.

There are several limitations of this study. First, registering the location of the most activated channel in fNIRS to the navigation rTMS system required a manual process using an optical digitizer by the operator. This overlapping process may be a possible source of inaccuracy in stimulating the exact “hot spot” determined by fNIRS. Nonetheless, the primary purpose of using fNIRS in this study was to find the hot spot, which was expected to have recovery potential in the lesional language associated area, so the problem of spatial accuracy was tolerable. Recently published MRI data-driven fNIRS mapping method (56) can be used in future studies to reduce inaccuracy, however, more studies are needed to address this problem. The second limitation of this study is the absence of the control group. In this study, all the patients received HF-rTMS in combination with the speech therapy without sham-controlled or speech therapy only group. Due to the lack of a control group, this study could not firmly conclude the effectiveness of lesional HF-rTMS on the most activated area. Finally, the small sample size of the study hinders us from drawing a decisive conclusion on the effect of the lesional HF-rTMS.

In conclusion, this study showed improvement of language function and changes of cortico–cortical interaction between the language-related areas after HF-rTMS on the most activated area determined with fNIRS in patients with poststroke with stable aphasia. Network analysis revealed an improved clustering coefficient in the left hemisphere with decreased global efficiency. Further randomized controlled trials with a larger sample size and the well-designed control groups using our protocol will provide more insights into the effect of HF-rTMS on the most activated area in the lesional hemisphere and the changes of corticocortical interaction after neuromodulation in the patients with stroke with aphasia.

## Data Availability Statement

The raw data supporting the conclusions of this article will be made available by the authors, without undue reservation.

## Ethics Statement

The studies involving human participants were reviewed and approved by Institutional Review Board of Seoul National University Bundang Hospital. The patients/participants provided their written informed consent to participate in this study.

## Author Contributions

WC interpreted the results and wrote the manuscript. JP and J-YL designed the study and recruited participants with W-SK and N-JP. SC and JL collected and analyzed the data. All the authors were involved in the data review process, provided feedback on the drafts, and have approved the final manuscript.

## Funding

This study was supported by the Basic Science Research Program through the National Research Foundation of Korea (NRF) funded by the Ministry of Science, ICT and Future Planning (NRF-2016R1A2B4013730) and the Seoul National University Bundang Hospital Research Fund (14-2015-0030).

## Conflict of Interest

The authors declare that the research was conducted in the absence of any commercial or financial relationships that could be construed as a potential conflict of interest.

## Publisher's Note

All claims expressed in this article are solely those of the authors and do not necessarily represent those of their affiliated organizations, or those of the publisher, the editors and the reviewers. Any product that may be evaluated in this article, or claim that may be made by its manufacturer, is not guaranteed or endorsed by the publisher.

## Supplementary Material

Supplementary Figure 1 Transcranial magnetic stimulation (TMS) and Navigation system. The system consists of a TMS chair, an optical digitizer, a Navigation computer, a TMS machine, and a figure-8 shape coil.

Supplementary Figure 2 T2-weighted FLAIR axial brain MRI images of patient number 2, showing large chronic infarction in the left fronto–temporo–parietal lobe. From left to right and top to bottom. R, right; L, left.

Supplementary Table 1 Subsection scores of K-WAB in 5 participants.
