# Supplementary figures and images for: Functional Network Changes After High-Frequency rTMS Over the Most Activated Speech-Related Area Combined With Speech Therapy in Chronic Stroke With Non-fluent Aphasia
Source: Front Neurol. 2022 Feb 10;13:690048. doi: 10.3389/fneur.2022.690048 (PMC8866644; doi:10.3389/fneur.2022.690048)

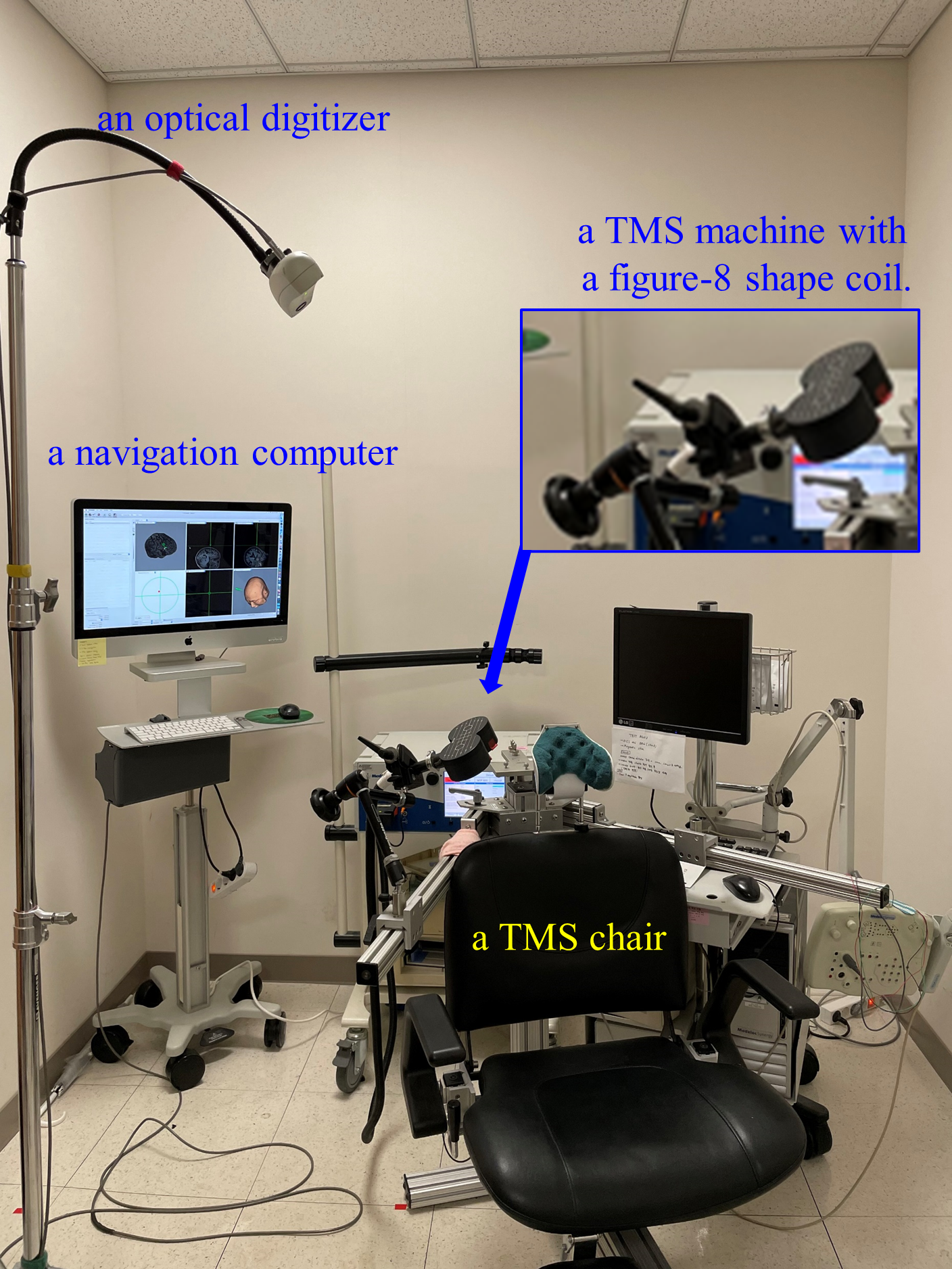

Supplement: Supplementary Figure 1 — Transcranial magnetic stimulation (TMS) and Navigation system. The system consists of a TMS chair, an optical digitizer, a Navigation computer, a TMS machine, and a figure-8 shape coil. [file Image_1.TIF]

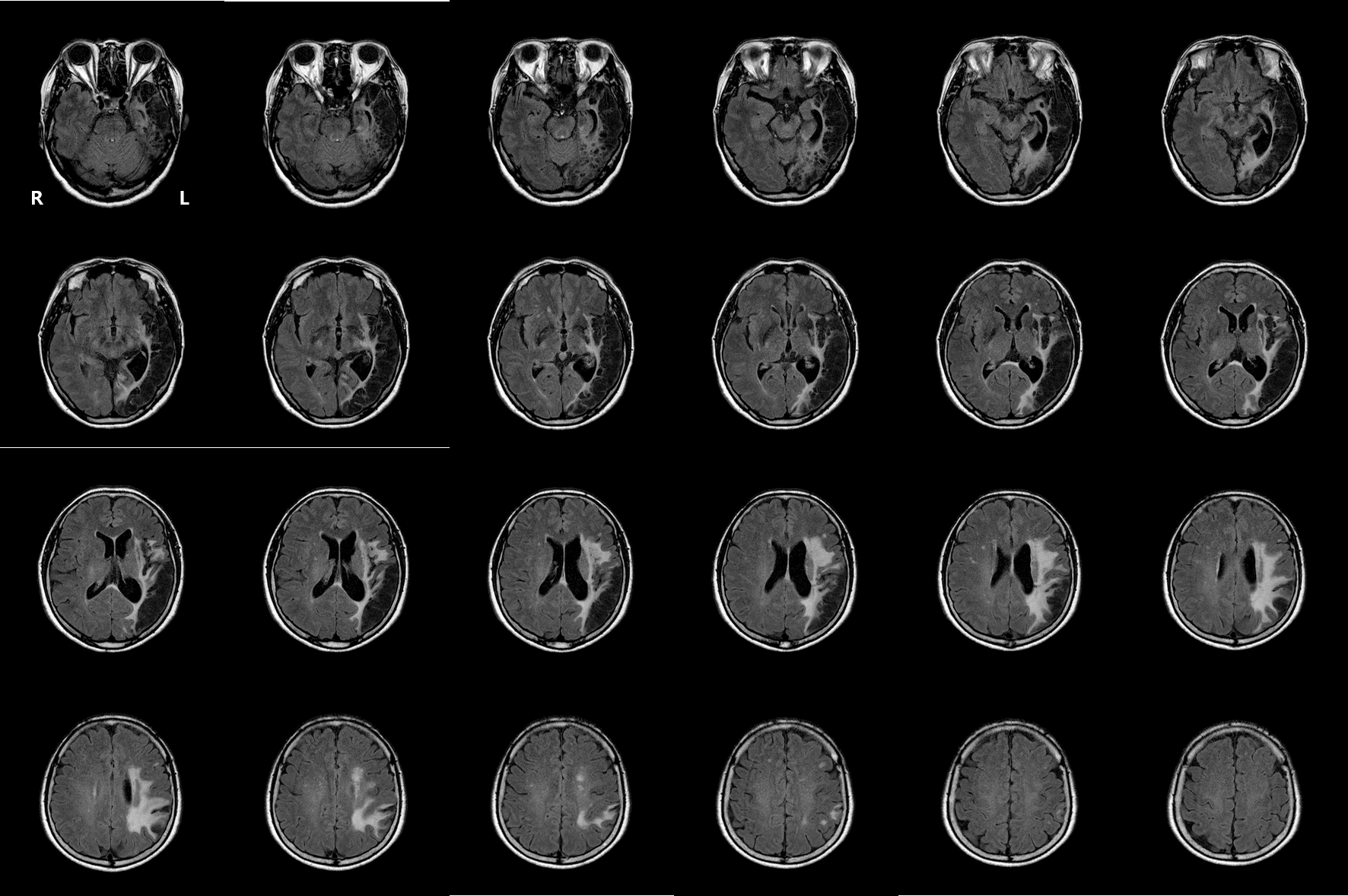

Supplement: Supplementary Figure 2 — T2-weighted FLAIR axial brain MRI images of patient number 2, showing large chronic infarction in the left fronto–temporo–parietal lobe. From left to right and top to bottom. R, right; L, left. [file Image_2.TIFF]
