# Supplementary material for: Functional Network Changes After High-Frequency rTMS Over the Most Activated Speech-Related Area Combined With Speech Therapy in Chronic Stroke With Non-fluent Aphasia
Source: Front Neurol. 2022 Feb 10;13:690048. doi: 10.3389/fneur.2022.690048 (PMC8866644; doi:10.3389/fneur.2022.690048)
Supplement: Supplementary Table 1 — Subsection scores of K-WAB in 5 participants. [file Table_1.docx]

**Supplementary TABLE 1** Subsection scores of K-WAB in 5 participants

| Patient number | 1 | 2 | 3 | 4 | 5 |
| --- | --- | --- | --- | --- | --- |
| K-WAB (T0) |  |  |  |  |  |
| Speech | 13 | 7 | 19.5 | 8 | 14.5 |
| Repeat | 16 | 16 | 100 | 60 | 22 |
| Reading | 59 | 45 | 100 | 29 | 54 |
| Comprehension | 142 | 89 | 197 | 96 | 146 |
| Naming | 43 | 7 | 94 | 51 | 78 |
| Writing | 45 | 18 | 99 | 28 | 36 |
| K-WAB (T1) |  |  |  |  |  |
| Speech | 14 | 9 | 19.5 | 9 | 14.5 |
| Repeat | 12 | 20 | 100 | 80 | 28 |
| Reading | 66 | 41 | 100 | 54 | 60 |
| Comprehension | 140 | 91 | 200 | 130 | 159 |
| Naming | 62 | 16 | 100 | 47 | 82 |
| Writing | 49 | 16 | 100 | 31 | 41 |
| K-WAB (T2) |  |  |  |  |  |
| Speech | 14 | 9.5 | 20 | 8 | 12.5 |
| Repeat | 22 | 24 | 100 | 86 | 34 |
| Reading | 65 | 39 | 100 | 50 | 65 |
| Comprehension | 152 | 116 | 200 | 126 | 148 |
| Naming | 40 | 15 | 95 | 49 | 62 |
| Writing | 47 | 13 | 100 | 30 | 32 |
